# Locating a novel autosomal recessive genetic variant in the cattle glucokinase gene using only WGS data from three cases and six carriers

**DOI:** 10.3389/fgene.2022.755693

**Published:** 2022-08-29

**Authors:** Geoffrey E. Pollott, Richard J. Piercy, Claire Massey, Mazdak Salavati, Zhangrui Cheng, D. Claire Wathes

**Affiliations:** ^1^ Department of Pathobiology and Population Sciences, The Royal Veterinary College, London, United Kingdom; ^2^ Comparative Neuromuscular Diseases Laboratory, Department of Clinical Sciences and Services, Royal Veterinary College, London, United Kingdom; ^3^ Department of Pathobiology and Population Sciences, The Royal Veterinary College, Hatfield, United Kingdom; ^4^ The Roslin Institute, Midlothian, United Kingdom

**Keywords:** cattle, glucokinase gene, recessive genetics, runs of homozigosity, WGS, Irish Moiled, perinatal mortality

## Abstract

New Mendelian genetic conditions, which adversely affect livestock, arise all the time. To manage them effectively, some methods need to be devised that are quick and accurate. Until recently, finding the causal genomic site of a new autosomal recessive genetic disease has required a two-stage approach using single-nucleotide polymorphism (SNP) chip genotyping to locate the region containing the new variant. This region is then explored using fine-mapping methods to locate the actual site of the new variant. This study explores bioinformatic methods that can be used to identify the causative variants of recessive genetic disorders with full penetrance with just nine whole genome-sequenced animals to simplify and expedite the process to a one-step procedure. Using whole genome sequencing of only three cases and six carriers, the site of a novel variant causing perinatal mortality in Irish moiled calves was located. Four methods were used to interrogate the variant call format (VCF) data file of these nine animals, they are genotype criteria (GCR), autozygosity-by-difference (ABD), variant prediction scoring, and registered SNP information. From more than nine million variants in the VCF file, only one site was identified by all four methods (Chr4: g.77173487A>T (ARS-UCD1.2 (GCF_002263795.1)). This site was a splice acceptor variant located in the glucokinase gene (*GCK*). It was verified on an independent sample of animals from the breed using genotyping by polymerase chain reaction at the candidate site and autozygosity-by-difference using SNP-chips. Both methods confirmed the candidate site. Investigation of the GCR method found that sites meeting the GCR were not evenly spread across the genome but concentrated in regions of long runs of homozygosity. Locating GCR sites was best performed using two carriers to every case, and the carriers should be distantly related to the cases, within the breed concerned. Fewer than 20 animals need to be sequenced when using the GCR and ABD methods together. The genomic site of novel autosomal recessive Mendelian genetic diseases can be located using fewer than 20 animals combined with two bioinformatic methods, autozygosity-by-difference, and genotype criteria. In many instances it may also be confirmed with variant prediction scoring. This should speed-up and simplify the management of new genetic diseases to a single-step process.

## Introduction

A review of 34 articles detailing work to map 38 novel autosomal recessive genetic conditions to their position on the genome (Pollott, 2018) suggested that finding the site of such a condition required the use of at least a two-stage methodology. First, the use of a suitable single-nucleotide polymorphism (SNP) chip in a case/control study to locate the *region* containing the new variant combined with a method searching for long runs of homozygosity (ROH), the more traditional chi-squared method being shown to be inadequate. The second stage used a range of “fine-mapping” methods to search within the highlighted region for the *site* of the new variant, many of which resulted in whole genome sequencing (WGS) of a few cases and controls. More recent methods have been developed, which use WGS as an initial step but such methods typically require additional resources or sequencing a large number of animals.

The objective of the current study was to see if it is possible to locate the position of a novel autosomal recessive genetic condition directly using the ideas contained in Pollott (2018) on WGS methodology and using a small number of cases and controls (in this case carriers) without recourse to more extensive resources, which may not always be available. To achieve this, we investigated combining this approach with a range of other bioinformatic tools and genetic ideas which may indicate the site of such a variant by reading the various signals in the WGS data. Using the variant call format (VCF; VCF (2019)) files from a suitable combination of cases and controls, it was suggested that typically about 16 animals would need to be sequenced in order to locate a new autosomal recessive variant using a “genotype criteria” approach (GCR; Pollott (2018)).

Whole genome sequencing is becoming more widely used to locate single novel variants with major effects, and a number of approaches have been used. In a large scale analysis of Holstein cattle WGSs, seven dominant conditions were located using the genome criteria approach (Bourneuf et al., 2017) involving one case for each of the seven conditions and a control population of 1,230 animals. The trio approach has been used by a number of authors (*see* for example Sayyab et al., 2016). This method takes WGSs of one affected offspring and its two parents and uses the genotype criteria method to find possible sites for the causative variant. The large number of sites identified is further reduced by a range of methods. Using one dog example (Sayyab et al., 2016), a filtering pipeline was established with seven steps, including genotype criteria and SIFT analysis, Sanger sequencing verification and sequencing of an additional 24 cases/controls. Runs of homozygosity methods have been widely used with SNP-chip data (Pollott, 2018) and Letko et al. (2020) report an example of using this method in Zwartbles sheep to locate a novel autosomal recessive condition associated with type 1 primary hyperoxaluria. Their study relied upon additional data from both the Sheep genomes project and 79 publicly available genomes of various breeds to provide “control” data for the GCR method. The methods reviewed here all required further data and analyses in order to locate the novel causative variant. In this article, we have tried to minimize this by looking for complementary methods, which can be used on the dataset alone.

Here, we test these ideas on a novel genetic disease found in Irish Moiled cattle. The Irish Moiled is an ancient hornless cattle breed native to the island of Ireland. It was popular in the 1800s, but by the late 1970s the pedigree herd numbered only 30 breeding females and two bulls. In 1979 the Rare Breeds Survival Trust recognized the Irish Moiled cattle as endangered and placed the breed on its “critical” list (Irish Moiled Cattle Society, 2020). The population size now numbers about 875 females and 90 bulls. Fortunately, novel fatal genetic diseases are relatively rare but when they do occur it is important to find the cause and implement plans to manage the condition *via* selective breeding, as soon as possible. A number of Irish Moiled cattle breeders were concerned about the seemingly high occurrence of early calf deaths in their herds. Affected calves had the following characteristics: days 1–2, the calf appeared slightly hyperactive with more playing/skipping than normal, and may have been seen drinking water from troughs or puddles, and also urinating more frequently than normal. Days 2–4, the calf started to deteriorate and became dull and lethargic. Days 4–6, the calf became dehydrated, very weak, and unable to stand. Death followed soon after. A 2–4 day-old calf was clinically very similar to a calf with septicemia; however, in contrast to septicemic calves, these calves did not respond to antibiotics and fluids given *via* an intravenous drip. Also when the blood glucose level of such calves was tested levels exceeded 30 mmol/L (Normal = 8 mmol/L). Breeders referred to this condition as “diabetes.”

An initial analysis was undertaken which suggested that there was likely a genetic basis to the disease (*see* Supplementary File Page S18), and since it was fatal, it could only be inherited as a recessive condition.

## Materials and methods

Throughout this article the ARS-UCD1.2 (GCF_002263795.1) build of the cattle genome was used and all chromosome positions quoted relate to it (Ensembl, 2020).

### Sample collection

Farmers were contacted *via* the Irish Moiled breed society and asked to submit hair samples for analysis. Clean tufts of tail hairs were plucked from either live cows/bulls or recently dead calves (within 8 h postmortem) by the animals’ owners. These were sealed in a paper envelope for posting to the laboratory. Information recorded included the sample type (bull, dam or dead calf); ear tag numbers of dam and sire; dam herd ID; calving date; calf sex; and time of calf death (stillborn/days after calving). DNA was then extracted from the hair follicles for processing as described in the Supplementary File (Page S2).

### Whole genome sequencing

Nine DNA samples were used in these analyses comprising three dead calves (cases) and six carriers (controls), which were either parents of the cases or parents of other dead calves (and grandparents of the cases) as illustrated in the pedigree (Supplementary Figure S3). These nine samples were sequenced on an Illumina NGS platform after sample preparation as described in Supplementary File (Page S2). Briefly, 1 µg of DNA per sample was processed using a TruSeq Nano DNA LT Library Prep Kit (Illumina, United States), according to the supplied protocol. This produced randomly sheared 350 bp inserts. After end repair and adapter ligation, DNA was amplified *via* polymerase chain reaction (PCR), and the product was purified using AMPure XP (Beckman Coulter, United Kingdom). Size-selected DNA from each animal was sequenced on the HiSeq machine to achieve 150 bp paired-end reads to cover the bovine genome with an average 30X coverage (>90 Gbp raw data with >85% Q30 (Phred-scaled)). Alignment, mapping, variant calling, and preparation of the final VCF file were carried out on the subsequent reads as described in Supplementary File (Page S2).

### Genotype criteria

Information derived from WGS data on a small sample of cases and parental carriers may contain a number of signals indicating the site of a novel autosomal recessive condition. “Across”-animal data should show a typical pattern of homozygous cases and heterozygous parental carriers at the candidate site; the “genotype criteria” approach (Pollott, 2018). Considering a single base position with a reference allele A for the given species and a new variant C which causes a novel autosomal recessive genetic disease, then the expected outcomes from matings between carriers in the population will be offspring with the genotypes AA, AC, and CC in the classical Mendelian ratio of 1:2:1. Lethals (CC) would be observed in the dataset if the effect of the new homozygous variant occurred after the time of recording the animal’s health status. The term “genotype criteria” (GCR) was used to mean the particular combination of case and control genotypes, which was required to indicate that a base position could harbor the novel lethal variant (Pollott, 2018). For example, in a dataset comprising five cases and 10 parental carriers, we would expect to find the novel lethal variant at a position showing CC genotypes in all five cases and a genotype containing the C allele in the 10 parental carriers, or all AC in the case of a biallelic position. The probability of occurrence of GCR under this condition would be 1/3^n^ in cases and 1/3^m^ in parental carriers, where *n* is the number of cases and m is the number of carriers that have been whole genome-sequenced (Pollott, 2018). If the VCF data file comprised 14 million positions (∼0.005 of the cattle genome), then a minimum of 15 animals would probably need to be genotyped in order to find one position with the required genotype criteria, that is, 1/3^15^ × 14 million = 0.98 (i.e. ∼1), the expected number of sites with the “correct” genotype criteria from the genome of 15 animals.

A script was written in Perl 5.28 to scan the final VCF file (containing all nine WGS animals) for the expected GCR pattern across cases and controls (i.e. all cases homozygous for the same allele and all controls heterozygous and containing this allele). In order to qualify for selection, a site had to have all genotypes with a Phred-scaled quality score greater than 12 and a depth of coverage more than 11 reads (Broad Institute, 2020). The identity of these sites was stored along with their relevant VCF record for later scanning and use.

### Autozygosity-by-difference and runs of homozygosity

“Within”-animal data should show long ROH around the new variant in cases, which are not present in controls. Variants causing a novel autosomal recessive genetic disease are expected to carry with them a very long haplotype originating from the animal in which the variant first arose. When a new case animal is formed then it contains two copies of this long haplotype, only broken up by any recombination events that have occurred since the formation of the original variant, and the new variant will be situated in a long ROH. This idea has been the basis for locating novel variants using SNP-chips for a number of years (*see* Pollott (2018) for a review) and can be used in a number of ways with VCF data. Long ROH throughout the genome could be found and one would expect to find the novel variant in the longest ROH in cases, possibly with adjustment for the situation in controls.

The autozygosity-by-difference (ABD) method measures runs of homozygosity on each chromosome of each individual in the dataset, cases, and controls, using genomewide single-nucleotide variant (SNV) (or SNP) genotypes (Pollott, 2018). Mean ROH length, in Kb, at each SNV positon is calculated for cases and controls separately and then their difference calculated as the ABD score. Missing genotypes were assumed to be homozygous reference allele. The likely site of the new variant is in the region with the greatest mean ROH in cases, after taking into account any breed-specific ROH found in controls, that is, the ABD score. The ABD method was programmed in Perl 5.28. The final VCF file was used as the basis to generate a file of sites as input to the ABD method. This method is sensitive to incorrectly called genotypes and so the VCF file was subjected to hard filtering as recommended by the Broad Institute (2020) in the absence of suitable databases to use for the recommended variant quality score recalibration (VQSR). A file of SNV were generated from the final VCF file, which passed the quality control tests shown in Supplementary Table S1, following a summary of the quality statistics of the VCF file (*see* Supplementary Figures S1, S2).

The ABD scores were used to look for the potential site of the novel variant causing calf mortality in the Irish Moiled dataset. The probability of each ABD score was tested using 100 permutations of the data based on the random allocation of animals to phenotypes and recalculation of the ABD scores (Pollott, 2018). Significance at the *p* < 0.01 level was considered as an indicator of a possible site of the new variant.

### Sorting intolerant from tolerant (SIFT) score

In a fatal genetic disease one would expect to find that the products from any change in the sequence would have a drastic effect on the phenotype of the animal so one could not only search for SNV with a potentially drastic effect but could also eliminate those with “silent” changes. The Variant Effect Predictor (VEP; McLaren et al., 2016) is a bioinformatic tool, which can take a change in a base at a given position on the genome and predict the outcome of that change on the corresponding coding or non-coding genomic feature. Using this method it is possible to model each base position in the VCF file to see the effect of the new variant on the phenotype in the form of a SIFT score (Ng and Henikoff, 2002).

The final VCF file from all the animals was annotated for variant effect prediction using Ensembl VEP command line v90.5 (McLaren et al., 2016), given the following flags: --tab --fork 8 --offline --species bos_taurus_merged. The VEP was used on all variant sites in the merged VCF file, and the results filtered for HIGH SIFT scores using VCF (Danecek et al., 2011). Various outcomes are given in the VEP but here the HIGH outcome was used for the SIFT score since this was a lethal variant. SIFT predicts whether an amino acid substitution affects protein function based on sequence homology and the physical properties of amino acids. The variant impact categories are subjective agreements between the VEP and SNPEff databases. However, high-impact variants are considered to have protein level disruption or change, while modifier or moderate variants impact non-coding regions of the genome. The SIFT score closer to zero is mostly represented by HIGH or modifier impact categories, while tolerated levels (SIFT score of 0.05–1) would show “minimal” to “no consequence” for the function of the genes under said variants. All sites with high-impact scores were captured in a separate file for further processing.

### Novel variants

The dbSNP database of NCBI (NCBI, 2019) contains data on variants already reported by researchers. Novel variants are unlikely to be contained in these datasets and so will not already have an RS number. Their absence may be another way to reduce the search area along the genome, as novel variants are likely to be found at sites that are not already logged in the relevant SNP database. Sites in the final VCF file that did not have an RS number were possible positions for the new variant. The VCF file was scanned for positions that did not have a previously allocated RS number, using a script written in Perl 5.28. Such sites were output for further analysis. The sites identified in this way were summarized by the number of genotypes containing the variant allele found at each site. Candidate sites contained a “potential” variant allele in all nine samples.

### Comparing datasets

Putting all these ideas together should make locating the site of the new variant on the genome possible using WGS data from a small number of animals without the need for any other data sources. The aforementioned analyses resulted in four independently derived sets of data, each of which could contain an indication of where the new variant might be found on the genome. These were *1*) the sites with the appropriate GCR, *2*) sites in long ROH, with a high ABD score significant at *p* < 0.01, *3*) sites with a high-impact SIFT score, and *4*) sites with no RS number. Each dataset was derived from the final VCF file by a method independent of the other three. If a site appears in all four datasets this is likely to be the site of the new variant. The four datasets were compared for overlapping positions by reading them into an Access database and linking on the site position.

### Predicting the effect of the novel variant

The SIFT scoring method described earlier is one method for modeling the effects of a new variant on the phenotype of the animal. PHYRE2 (Kelley et al., 2015) is an alternative approach, which searches for homologous sequences in a database of known proteins. The reference sequence and its equivalent using the new variant were entered into the PHYRE2 database to see the effect of the site of the proposed new variant on amino acid sequence and protein structure.

### Methods used to confirm the likely base position of the variant

Two independent methods were used in an attempt to confirm the site derived from the methods used on the WGS data described earlier. A sample of Irish Moiled animals comprising three bulls, 42 cows, and 18 dead calves (male and female) were genotyped using SNP-chips. The DNA from these animals were then analyzed using *1*) genotyping by PCR at the suggested site and *2*) the ABD method on the SNP-chip data.

#### SNP processing

DNA samples extracted as described in the Supplementary File (Page S2) were genotyped in the Department of Pathology, University of Cambridge (United Kingdom) and Gen-Probe (Heron House, Oaks Business Park, Crewe Road, Wythenshawe, Manchester, M23 9HZ, United Kingdom) using either *1*) the Illumina BovineSNP50 BeadChip (Version 1, Illumina Inc., San Diego, CA, United States) (50k SNP, *n* = 17); *2*) the BovineHD Genotyping BeadChip (777k SNP, *n* = 68), or *3*) both chips (*n* = 7).

The SNP genotypes were prepared for all subsequent analyses using PLINK 1.9 (Purcell et al., 2007; Chang et al., 2015; PLINK, 2017). Quality control parameters were used to edit the data. This involved setting a lower limit on both sample and SNP quality at a call rate greater than 90%, and SNPs were retained in the dataset if they were in Hardy–Weinberg equilibrium. This was determined using Fisher’s Exact Test, with a probability threshold of 0.05 and using the mid-p adjustment described in Graffelman and Moreno, 2013. The latest SNP positions were updated to ARS-UCD1.2 (GCF_002263795.1) build of the bovine genome using SNPchiMp (Nicolazzi et al., 2014; Nicolazzi et al., 2015). In addition, a merged set of data was produced using SNPchiMp, combining all genotyped animals from both the LD and HD datasets with common SNP. This merged dataset was then used in KING (Manichaikul et al., 2010) to generate relatedness coefficients between all genotyped animals, based on whole genome SNP genotypes and to aid pedigree checking. Any animal whose pedigree did not match the relatedness information from the SNP data was discarded. In all 13 animals were discarded for both pedigree and quality control reasons. Because nine animals were also used for the WGS analysis, they too were excluded from the SNP ABD analysis, in order to produce a dataset of independent animals.

#### Autozygosity-by-difference

The ABD method (Pollott, 2018), described earlier, was used on the merged SNP-chip dataset. The probability of each SNP ABD score (difference between mean ROH length (Kb) from cases and controls at each SNP position) was tested using 1,000 permutations of the dataset based on random allocation of animals to phenotypes and recalculation of the ABD scores. Significance at the *p* < 0.001 level was considered as an indicator of a possible site of the new variant.

#### Genotyping by PCR analysis

Primers (5′-CAT​GAA​CCC​AGT​GTC​ACA​GC-3′ and 5′-CTC​TCC​GTG​GAA​GAG​CAG​AT-3′) were designed using Primer3 (version 4.1.0; http://primer3.ut.ee) to amplify a 218 bp product spanning the identified variant locus. The primer design was based on the published sequence for the *Bos taurus* (UMD3.1; GCF_000003055.6) glucokinase gene ENSBTAG00000032288. Exon/intron boundaries were derived from this in combination with mRNA RefSeq NM_001102302. PCR was performed using AmpliTaq Gold polymerase (Applied Biosystems), according to the manufacturer’s protocol. Products were purified using the QIAquick PCR purification kit (Qiagen) and sequenced by Sanger sequencing using the forward and reverse primers. Sequence analysis was carried out in CLC Genomics Workbench (Qiagen, 2013). The candidate site was updated to the ARS-UCD1.2 (GCF_002263795.1) genome build using the UCSC Genome LiftOver facility (UCSC, 2020).

## Results

The WGS data from all nine animals, three cases, and six carriers, resulted in a final VCF file comprising 8,234,367 biallelic autosomal single-nucleotide variants, which were to be used for all subsequent WGS analyses. These are summarized by chromosome in Table 1 along with the length of each chromosome aligned in ARS-UCD1.2 (GCF_002263795.1) build of the cattle genome.

**TABLE 1:** Summary of results by chromosome.

BTA	Length (bp)	Number of SNV in VCF file	Number of indels in VCF file	Estimated number of GCR	Actual number of GCR	Number of SIFT sites	Number of NoRS9 sites
1	158,534,110	535,135	101,947	32	582	251	694
2	136,231,102	452,534	82,255	27	4	279	165
3	121,005,158	394,128	70,162	24	0	383	271
4	120,000,601	373,759	72,506	23	22	485	347
5	120,089,316	401,056	71,836	24	2	458	173
6	117,806,340	362,930	71,311	22	4	174	221
7	110,682,743	358,947	67,655	22	2	491	250
8	113,319,770	319,261	62,634	19	3	204	486
9	105,454,467	328,559	62,502	20	2	179	185
10	103,308,737	353,791	63,775	21	1	293	162
11	106,982,474	324,386	58,178	19	9	337	134
12	87,216,183	335,302	63,750	20	21	139	148
13	83,472,345	232,616	43,728	14	2	249	188
14	82,403,003	262,990	49,112	16	1	108	135
15	85,007,780	285,748	54,791	17	2	374	160
16	81,013,979	268,622	49,356	16	0	225	388
17	73,167,244	278,406	50,191	17	5	198	108
18	65,820,629	194,623	37,810	12	0	522	384
19	63,449,741	227,124	39,664	14	0	449	110
20	71,974,595	230,092	43,456	14	1	110	84
21	69,862,954	202,249	38,102	12	2	205	261
22	60,773,035	180,922	33,961	11	1	147	164
23	52,498,615	255,467	42,220	15	58	566	337
24	62,317,253	228,628	39,037	14	3	83	109
25	42,350,435	142,293	25,212	9	0	225	43
26	51,992,305	177,147	33,190	11	1	133	120
27	45,612,108	175,471	32,421	11	1	99	88
28	45,940,150	179,521	31,214	11	1	92	99
29	51,098,607	172,660	31,952	10	0	306	358
Total	2,489,385,779	8,234,367	1,523,928	496	730	7,764	6,372

BTA, chromosome number; GCR, genotype criteria sites; NoRS9, number of sites with no RS number and with at least one alternate allele in all nine genotypes. “Estimated number of GCR sites” assumes an even spread across the genome. “Number of SIFT sites” was the number of sites with a “HIGH” SIFT score.

### Genotype criteria

Searching the final VCF file for sites with the appropriate genotype criteria (all homozygous cases for the same genotype and all carriers heterozygous containing one allele forming the homozygote in cases) resulted in the identification of 730 sites. These are shown broken down by chromosome in Table 1. Applying the formula, 1/3^n^ cases and 1/3^m^ parental carriers to over 9 million SNV and indels, we would expect to find ∼496 sites fitting the genotype criteria. There were clearly more GCR sites than expected in this set of animals. Chromosomes 1 and 23 appeared to have more sites than expected (Table 1).

### Autozygosity-by-difference method

**FIGURE 1:**
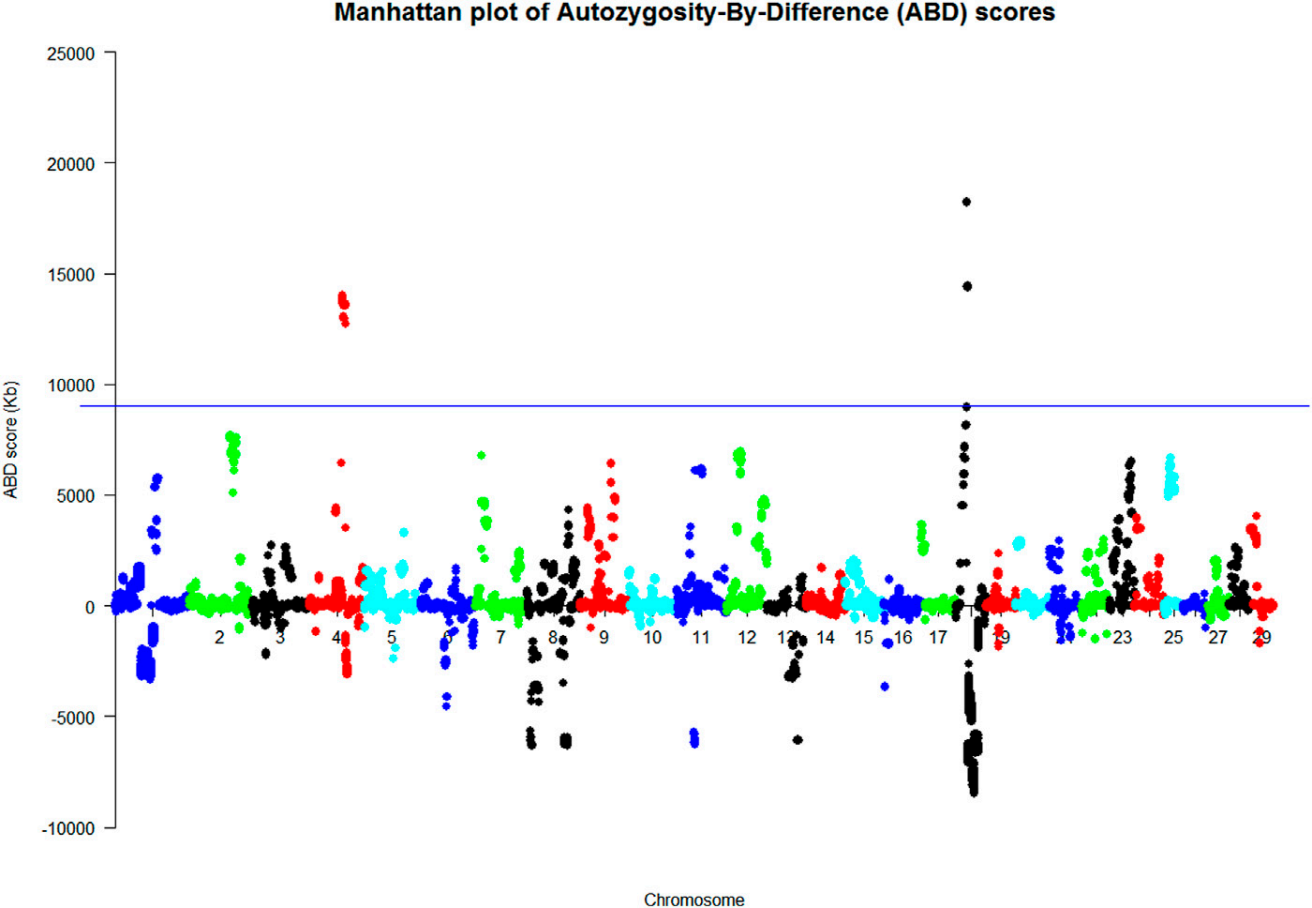
Manhattan plots of the ABD analysis of nine WGS animals (Kb). *p* < 0.01 at ABD score = 9,034 Kb where the ABD score on the *y* axis was the difference between the mean length of cases minus that of controls at each site.

In order to run the ABD method on the final VCF file, the hard-filtering criteria shown in Supplementary Table S1 were used on the extracted biallelic SNVs for the dataset. This resulted in a file of 629,716 SNV for the ABD analysis. ABD software was used to generate the Manhattan plots shown in Figure 1 and Supplementary Figure S4. The two plots in S4 show the mean ROH length at each of the base positions in the VCF file for cases and carriers, respectively, while Figure 1, the ABD score, shows the difference between them. Long ROH were found on BTA4 and BTA18. Probabilities for the ABD scores were generated from 100 permutations of the dataset, and the regions of the genome with *p* < 0.01 are summarized in Supplementary Table S2. The 0.01 probability level was computed to be at an ABD score of 9,034 Kb. Supplementary Table S2 shows that the length of BTA4 above the 0.01 probability threshold was 7.804 Mb. The highest mean ROH length in cases was 14.197 Mb so the long ROH found on BTA4 continued on either side of the significant region. Similarly on BTA18, the highest mean ROH score in cases was 22.4 Mb long.

### SIFT score

Using the VEP to estimate the effect of each of the SNVs and indels in the final VCF file resulted in 65,961 records of HIGH impact SNVs located at 7,764 different autosomal positions. The distribution of these sites is summarized by chromosome in Table 1.

### Sites with no RS number

The VCF file contained 340,893 sites with no RS number. Only 11% (6,372) of the sites had genotypes, other than the homozygous reference genome, in all nine cases and carriers (NoRS9). The breakdown of these by chromosome is summarized in Table 1. These are likely to contain the novel variant.

### Overlap of GCR, ABD, NoRS9, and SIFT results

So far, four possible datasets were generated that might contain the site of the novel variant causing this new autosomal recessive condition. The overlap between the four datasets is summarized in Table 2. The genotype criteria method resulted in the fewest sites identified (730), with the other methods increasing in the order autozygosity-by-difference, no RS number with nine genotypes, and high SIFT score. Combining the GCR method with each of the others in turn allowed the identification of 22 (ABD), 1 (HIGH SIFT), and 78 (NoRS9) sites in common. One site appeared in all four datasets located at position Chr4: g.77173487A>T (ARS-UCD1.2 (GCF_002263795.1). We may tentatively conclude that this is the site of the novel variant, causing early calf death in the Irish moiled breed.

**TABLE 2:** Overlap between the four methods for locating a likely novel variant site.

Method	Genotype criteria (GCR)	Autozygosity-by-difference (ABD)	High-impact SIFT score (SIFT)	No registered SNP number (NoRS9)
GCR	**730 (22)**			
ABD	22 (10)	**896 (635)**		
SIFT	1 (1)	12 (12)	**7,764 (12)**	
NoRS9	78 (8)	41 (40)	8 (1)	**6,372 (40)**
GCR+ABD			1 (1)	8 (8)
GCR+SIFT				1 (1)
ABD+SIFT				1 (1)
GCR+ABD+SIFT				1 (1)

The table shows the number of sites in the final VCF file identified by each method (numbers in the BTA4 high-ABD region shown in parentheses).

### PHYRE2 prediction

The PHYRE2 prediction (Kelley et al., 2015) of secondary structures between the reference genome and new variant *GCK* model at the beginning of exon 8, which contains the possible site of the new variant, Chr4: g.77173487A>T (ARS-UCD1.2 (GCF_002263795.1), predicted the amino acid sequence NPGQQLWY from the reference genome being changed to NPGQQLLY with the new variant.

### Independent confirmation of the results

#### Sanger sequencing

A sample of 41 animals was Sanger sequenced at position Chr4: g.77173487A>T (ARS-UCD1.2 (GCF_002263795.1)), following PCR of the region surrounding this site. Table 3 shows the Fisher’s exact test (Fisher, 1922) results (Freeman and Halton. (1951)) for animals falling into three categories; calves, carriers, and live animals of the unknown status by three genotypes.

**TABLE 3:** Animal status by genotype for the 41 Sanger-sequenced animals at Chr4: g.77173487A>T (ARS-UCD1.2 (GCF_002263795.1)).

Animal status	AA	AT	TT	Total
Calves	4	2	7	13
Known adult carriers (live)	0	6	0	6
Status unknown adults (live)	13	9	0	22
Total	17	17	7	41

Table 3 shows that all TT animals were calves, all of which died of the symptoms described earlier. All live animals were either AA or AT. The overall results were significant with a probability = 1.868e−05 (0.00001868) for this 3 × 3 table arising by chance, thus indicating a likely association between genotype and health status at this site.

#### ABD method based on genotypes derived from the PCR analysis

**FIGURE 2:**
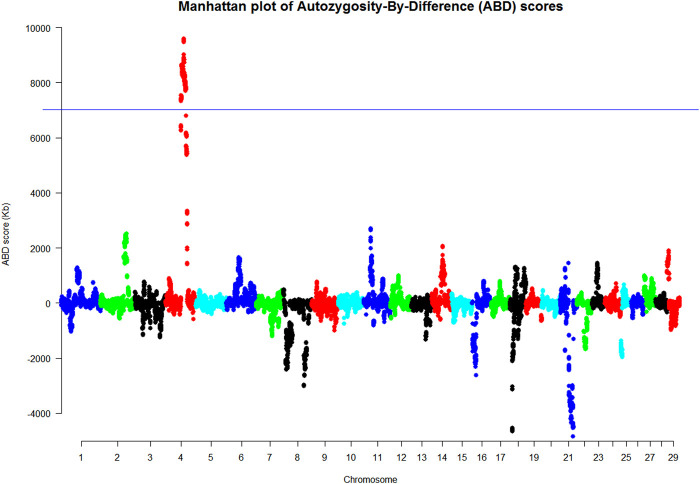
Manhattan plot of the ABD analysis of the SNP-chip analysis based on the genotypes found in the PCR analysis (Kb). These results were based on animals with phenotyping informed by the PCR results (*p* < 0.001 at ABD score = 7,023 Kb where the ABD score on the *y* axis was the difference between the mean length of cases minus that of controls at each site).

The merged dataset (HD and LD chip data merged using SNPchiMp) comprised 63 animals (19 cases and 44 controls) and 42,453 SNPs after quality control conditions were met. The 42 animals used in the PCR analysis were selected for the ABD analysis, which excluded the WGS animals so that this analysis was independent of the WGS ABD analysis. The animals were allocated to their case/control status based on their PCR genotype at the highlighted location. The results of the ABD analyses are summarized in Figure 2 and Supplementary Figure S5 and showed a 20.8 Mb length of BTA4 with a permuted probability <0.001, from 1,000 permutations, equivalent to an ABD score greater than 7,023 Kb. This region was from position Chr4: g. 62872037 to g. 83635054 (ARS-UCD1.2 (GCF_002263795.1)), which includes the site highlighted as the putative causal variant from the WGS analyses.

The results in Supplementary Figure S5 show a long ROH on BTA21 but this was present in both the case and control animals, which negated each other in the ABD score analysis. This is a good example of the benefit of the ABD method. Also, the long ROH found in WGS cases on BTA18 (Figure 1) was not a feature of this larger set of results. There was reduced variability of these results with a higher number of animals compared to those from the WGS dataset analysis with only nine animals.

## Discussion

This work had two objectives. One general and the other more specific. The generally applied objective was to test the idea that it is possible to find the site of a novel autosomal recessive variant using just a small number of whole genome-sequenced animals and appropriate bioinformatic methods, thus circumventing the need for the commonly-used two-stage approach highlighted by the review of Pollott (2018) or the collection and/or use of further data. The specific objective was to find the site of a new autosomal recessive condition thought to exist in Irish Moiled cattle.

### Bioinformatic methods used with WGS data to find the site of a new autosomal recessive variant using a small number of cases and controls

In the current study, four bioinformatic methods were tested to try to find the location of the new variant causing early calf death in the nine Irish Moiled animals and relied on the “correct” site appearing in all four methods. No additional data from the Irish Moiled or any other breeds were used. Two of the methods (GCR and ABD) do not require any prior information about genes, SNP, or other genomic features but rely on across- and within-animal patterns of information contained in the genotypes at each SNV/indel found in the VCF file. Ideally one would like to use these two methods alone since they are not only independent of any prior knowledge about genome features (except the reference genome for the alignments and generation of the VCF file) but they will also be able to find a new variant causing an autosomal recessive condition anywhere on the genome, even when located outside a protein-coding region: a useful feature of the two methods. As has been seen, using three cases and six controls with the ABD method and GCR combined revealed 10 possible sites in a 7.795 Gb stretch of Chr4 between g.70889821 and g.78684588 (ARS-UCD1.2 (GCF_002263795.1)), involving 18,705 SNVs. The underlying implication of this approach is that with more animals, either cases or controls, we would find fewer sites and so make the search considerably more straightforward and find just a single causative variant site.

It is worth noting that when using WGS methods, the depth of coverage can have an important effect on the results and is related to the costs of genotyping. In this article, a 30X coverage of the genome was used. In a study looking at the relationship between depth of coverage and the ability of an experiment to locate novel variants, Jiang et al. (2019) found 10X to be an ideal balance between cost and accuracy. Not suprisingly, the higher the depth of coverage, the greater is the ability to discover novel variants.

#### The genotype criteria approach

The method used to find the sites meeting the genotype criteria was based on a number of implied assumptions not stated by Pollott (2018). First, GCR sites would be evenly distributed across the genome. Second, the higher the number of animals used the greater the chances of finding the GCR site of the new variant. Third, a GCR site was not dependent on the balance of cases and controls in the samples. Fourth, the location of a single GCR site was independent of the genetic relationship between cases and controls. Each assumption was tested using the data analyzed in this study, either the final VCF file for BTA4 or the SNP-chip data with phenotypes allocated by the PCR results as appropriate. The detail of these investigations is given in the Supplementary File (Pages S10–S17).

##### Evenly spaced GCR sites across the genome

The results in Table 1 show that some chromosomes contain no GCR candidate sites at all (BTAs 3, 16, 18, 19, 25, and 29). Many chromosomes contained far fewer GCR sites than expected whereas others contained a much greater number than expected (BTAs 1 and 23). A GCR site (in this case) comprises two components; the 0/1 in all controls and the 1/1 in all cases (using 0 to mean the reference allele and 1 the new or alternative variant). We might expect the chromosomes containing long ROH in cases potentially to have many more GCR sites than others. Inspection of Supplementary Figure S4 shows BTA4 and BTA18 as having the longest mean ROH but only BTA1 had a large excess of GCR sites. Using the information shown in Supplementary File, the original implied assumption from Pollott (2018) of an even distribution of GCR sites across the genome has not been verified here, and this has implications for the number of animals required to find a new variant site using WGS data alone. Clearly, the long ROH on BTA4 were linked to a large number of GCR sites but that was not so on BTA18.

##### Finding a causative variant

The second assumption about the use of GCR sites to locate a novel autosomal recessive variant was that the greater the number of animals that are genotyped, the better the chance of locating the new variant. It appears, from the work reported in Supplementary File, that the number of genotyped animals required to find the single candidate sites is three to four times greater than the predicted original formula of Pollott (2018); one case and 29 controls appears to require the fewest total animals genotyped to find the single candidate site in the SNP-chip dataset used. However, this “long tail” is due to several GCR sites being close together around the candidate site and always being “found” in the generated datasets. Differentiating between them may require another method or using a different set of controls, perhaps from more distantly related individuals.

##### The balance of cases and controls

The basic calculation of the number of animals required to find a GCR site is independent of the balance between the number of cases and controls used. Supplementary Figure S10 demonstrates that the lower the number of cases used, the fewer the total number of animals required to be genotyped. At first sight, these are rather startling results. However, outside the candidate site, it is much more unlikely to find all controls with a heterozygote genotype, whereas there will be many sites with all homozygous genotypes in cases; after all long ROH imply many 1/1 genotypes and so more sites potentially could meet the genotype criteria. The information in the Supplementary File (pages S10–S16) clearly demonstrates that the number of animals required is much closer to the theoretical numbers when large ROH regions are excluded from the analyses. The minimum number of animals required to find the candidate site is still slightly greater than the theoretical figure but this may be due to some other small areas of ROH not removed from the dataset. There are an enduring number of GCR sites in the high-ROH regions, which inflate the results in contrast to the theoretical number of sites expected. This illustrates why theory and “practice” may differ.

##### The genetic relationship between cases and controls

The nine animals used in the WGS analysis comprised three cases and six parental controls. In order to investigate whether the closely related controls might inflate the number of GCR sites found, an alternative SNP-chip dataset was derived using controls that were most distantly related to the cases (Supplementary File and Supplementary Figure S12). In this case, the number of animals required to find the new variant site was much closer to the theoretical expectation than with the parental controls.

#### The autozygosity-by-difference method

The ABD method was developed originally to locate regions of the genome likely to contain a new autosomal recessive variant using SNP data (*see* for example Posbergh et al., 2018). In the current analysis, it has been applied to WGS data from animals for the first time, as well as being used to confirm the results in an independent sample of SNP-genotyped animals. Because the method is sensitive to incorrectly called genotypes, a feature of WGS data, it was necessary to employ hard filtering criteria (*see* Supplementary Table S1) of both sites and genotypes in order to get a useable set of data. In this case, the VCF file was reduced from ∼12 million to ∼630,000 sites but this would differ under alternative hard-filtering criteria. Since VQSR methods were not available in the current situation (due to the species and number of animals genotyped) an alternative approach was taken; selecting sites and genotypes to fall within ±2 s.d. of the mean (or peaks in the case of bimodal variables). This allowed the location of several long ROH, one of which was found, by additional methods, to contain the new variant.

As well as using alternative hard-filtering criteria, it may be possible to use other approaches, including site sampling or sliding windows, to locate the region containing the new autosomal recessive variant. Using a site-sampling approach with a VCF file one could randomly select, say, 5–10% of sites evenly spread across the genome with the ABD method. Repeated samples of these SNVs, (say 100), could be randomly drawn and the 100 sets of ABD results averaged at each site. Alternatively, one could use a sliding window of, say, 10,000 base positions and count the number of homozygous variant case and control genotypes in each window. The window would then be moved along the genome at a given interval, say every 1,000 base positions, and the results plotted. One would expect to find the new variant causing the autosomal recessive condition in the region with the highest ABD-type score. Both these methods would overcome the problem of a single incorrectly called genotype disrupting the long ROH in the ABD results and the need for hard filtering.

Using ABD on the hard-filtered WGS data resulted in the identification of two regions of the genome having an ABD score above the 0.01 probability threshold, and therefore likely to contain the new variant (Figure 1). Two aspects of Figure 2 and Supplementary Figure S4 are of note. First, there were a number of long ROH found in the controls throughout the genome with BTA18 having the largest mean ROH length. This was also found in the cases, but the effect of combining the two sets of data in the ABD score was to remove many of these “breed-specific” ROH and leave those which probably harbored the new variant. This is one of the advantages of the ABD method, particularly in rare breeds, but it has also been shown to be effective in removing long breed-specific ROH in studies of new variants in the myostatin gene in both Texel sheep (Pollott, 2013) and Piedmontese cattle (Biscarini et al., 2013).

The ABD method was also used for another purpose in this work; to confirm the WGS results on an independent set of animals with SNP-chip-derived data (Figure 2). As with the WGS ABD results, there were many long ROH in the SNP-chip dataset in both cases and controls. In this instance, there were two very long ROH on BTA4 and BTA21, but interestingly not BTA18 as was found in the WGS data. The smaller number of animals used in the WGS analysis probably resulted in BTA18 having a long ROH due to sampling of closely related individuals. Supplementary Figure S5 also shows BTA18 to have a long ROH but it was less pronounced in this larger dataset. In Figure 2, the result of subtracting the control ROHs from that of cases at each site reduced the noise considerably and left BTA4 as the only significant peak by a considerable margin. Once again it has been demonstrated that the power of the ABD method to remove noise works effectively to highlight the region containing the potential causal variant.

#### GCR, ABD, SIFT score, and RS number

The approach in this work has been to use several bioinformatic methods on WGS data to see if they can pinpoint the site of a causal variant of a new autosomal recessive condition. Table 2 has highlighted a significant region on BTA4 using the ABD method that contained 12 sites meeting the genotype criteria. The earlier discussion has suggested that using more animals may have reduced the number of candidate sites by a small amount, but a greater use of unrelated controls may have reduced the number of GCR sites in the target area more effectively.

In this set of results, the SIFT scores were the crucial factor in determining the suggested site of the new variant. Position Chr4: g.77173487A>T (ARS-UCD1.2 (GCF_002263795.1) was the only one of the 22 GCR sites in the target area to have a high-impact SIFT score (0–0.05). However, only sites in or near a coding region are scored using the SIFT method so it is not always going to find the causative site if it is located outside these regions of the genome.

The use of the absence of an RS number could be useful but, in this case, did not prove to be the final factor locating the novel variant site.

### Other types of inheritance and effects

This article reports the search for a new autosomal recessive variant causing a fatal condition in calves using a range of bioinformatic methods. It raises questions relating to whether the methods would work with autosomal recessive conditions with differing phenotypes or with other modes of inheritance.

In this example, the phenotype was mortality in early postnatal life. This had the advantage of being an obvious phenotype. As the deaths occurred on widely scattered farms it was, however, not possible in this instance to collect suitable pre- or postmortem samples for follow-up analyses. A new non-fatal condition may be less easy to identify initially but there are likely to be more opportunities to collect appropriate samples to confirm phenotype diagnosis.

Having suggested this WGS approach for finding a new autosomal recessive variant, the question arises about its general usefulness with variants involving other modes of inheritance. A dominance mode of inheritance can be thought of as the reverse of the recessive mode. One would expect to find cases to be 0/1 or 1/1 and controls to be 0/0 so the genotype criteria would be different compared to the recessive mode of inheritance. However, the number of animals required to use the GCR method would be very similar with cases being 2/(3^n^) and controls 1/(3^m^); the numerator having little effect with such a large denominator. The ABD method could only be used if all cases were 1/1 but that is unlikely with a dominant condition due to the large number of heterozygotes likely to be in the population. Alternatively, if there was some way to phenotypically distinguish 1/1 from 1/0 cases this would be useful. The 0/0 controls are unlikely to be situated in long ROH since they are likely to have been subjected to many generations of recombination, so alternative methods may be required. The new dominant variant would be situated in a long haplotype so it may be possible to adapt haplotype discovery methods to this situation. Both the SIFT score and RS number methods would be applicable but they are less powerful than the other two because they rely on previous knowledge and, in the case of SIFT, it only works for a limited distance around a protein-coding region.

These methods could be used for a recessive sex-linked new variant, that is, one found on the X chromosome. Males would provide no useful data in this case so only females would be required. Both the ABD and the GCR methods would work the same way but with a lot fewer sites to search (only the X chromosome data would be needed).

### Finding the causative variant for a perinatal mortality syndrome in Irish Moiled cattle

The likely site for the causative variant of this fatal perinatal condition in Irish Moiled animals has been successfully located using just six parental control animals and three cases. Perinatal mortality (within 24 h of birth) typically occurs in about 6–10% of calves born (Brickell et al., 2009), with a further 3–4% dying in their first month, mainly from infectious disease (Johnson et al., 2017). The site highlighted at Chr4: g.77173487A>T (ARS-UCD1.2 (GCF_002263795.1) was located in the glucokinase gene (*GCK*) and is a splice acceptor variant. Analysis of the OMIA website (Nicholas and Hobbs, 2013; OMIA, 2020) showed splice acceptor variants to be responsible for ∼8% of known variants in non-laboratory animals. There was a clear difference in the PHYRE2 prediction of the secondary structures between the reference genome and new variant *GCK* model. As observed from the SIFT score results, this is expected to have a disruptive effect on the operation of the *GCK* gene.

Glukokinase is a key enzyme found in the liver, pancreas, brain, and endocrine cells of the gut. It catalyzes the starting point of glycolysis by phosphorylating glucose to form glucose-6-phosphate (Matschinsky et al., 1993). The crystal structure has revealed that glucose binds in a deep cleft between a large and small domain of GCK, resulting in a conformational change and enzyme activation (Kamata et al., 2004). Glucokinase stimulates glucose uptake, glycolysis and glycogen synthesis by hepatocytes, whereas in pancreatic β-cells it plays a crucial role in glucose-stimulated insulin secretion. Glucose homeostasis is essential in mammals and is under tight endocrine control, with insulin acting as the key regulator.

There are currently 922 SNPs listed within the bovine *GCK* gene (NCBI, 2019) but the closest to the new variant site flanking either side were at Chr4: g.77173441 (an intron variant) and Chr4: g.77174392 (ARS-UCD1.2 (GCF_002263795.1)), some 46 and 905 bp away, respectively. A segment of the ARS-UCD1.2 (GCF_002263795.1) genome 30 bp either side of the candidate variant was selected and BLASTn (Altschul et al., 1990) was used with the 61 bp sequence to find any homologous region on the human genome. A 40 bp length of sequence was found with 35 identical bases and a score of 50.9 bits (55) and no gaps. This was located on the reverse strand of human Chr7: g.44146590 to g.44146629 (GRCh38.p13 (GCF_000001405.39), in the *GCK* gene. The location on the human genome equivalent to the candidate variant found in Irish Moiled calves was at Chr7: g.44146620 (GRCh38.p13 (GCF_000001405.39). This was a highly conserved site with 96 out of the 100 vertebrate genomes shown on the UCSC (UCSC, 2020) genome browser, all having a T on the forward strand, the remaining four being not reported. No SNP was found at this site in the human database but there was an SNP reported at the adjacent position (Chr7: g.44146619; GRCh38.p13 (GCF_000001405.39)), which was cataloged as rs1167675604, a C>T change on the forward strand. This site was also highly conserved in 96 out of the 100 vertebrate genomes on the UCSC genome browser and was also a splice site acceptor variant. The ClinVar (ClinVar, 2020) record for this variant states that “the variant disrupts a canonical splice site and is therefore predicted to result in the loss of a functional protein, found in at least one symptomatic patient, and not found in general population data.” Its incidence was estimated to be well below 0.001% of the population. In addition, the Varsome (Varsome, 2020) record for this SNP states that the effect of the variant was “Very Strong,” which means “Null variant (intronic within ±2 of splice site) affecting gene *GCK*, which is a known mechanism of disease (gene has 378 known pathogenic variants, which is greater than minimum of 3), associated with diabetes mellitus, permanent neonatal 1, maturity onset diabetes of the young, type 2, and hyperinsulinemic hypoglycemia, familial 3.”

The mouse genome was also investigated in the same way but no SNPs were found in the candidate region.

Over 600 variants have been reported in the human *GCK* gene, which have varying effects depending on their location (Osbak et al., 2009; OMIM 138079). Heterozygous inactivating variants cause a condition known as maturity onset diabetes of the young, characterized by mild fasting hyperglycaemia. Homozygotes are much rarer in the human population, and neonates present earlier with permanent neonatal diabetes mellitus. In mice, however, pups born with global *GCK* knockout (−/−) are slightly smaller than wild-type animals (+/+), have glucose levels about eight-fold higher and die within 3–5 days (Grupe et al., 1995). Tissue specific β-cell knockouts die within 4 days of birth, whereas hepatic knockout impairs glucose utilization and glycogen synthesis but with only mild hyperglycaemia (Postic et al., 1999).

Pregnancy outcome in women depends on a combination of the genotype of both mother and fetus (Spyer et al., 2001). When the fetus carries a single *GCK* variant, this affects glucose homeostasis with reduced insulin secretion, so both placental and birth weight are reduced (Hattersley et al., 1998; Spyer et al., 2008). During pregnancy, the fetal glucose supply is derived almost entirely from the dam across the placenta using facilitated diffusion by glucose transporters. In ruminants, this uptake is regulated sequentially by GLUT1 and GLUT3 (SLC2A1 and SLC2A3) (Wooding et al., 2005).

The fetus has a low capacity for endogenous glucose production but this increases in late gestation, in response to the pre-term increase in glucocorticoid production, together with catecholamine and thyroid hormone stimulation. These promote hepatic glycogen synthesis and gluconeogenesis, which are essential in providing the neonatal calf with an adequate glucose supply as milk lactose on its own is insufficient (Hammon et al., 2013). The postnatal maturation in the regulation of energy supply may thus explain why lack of GCK activity is fatal at this stage of life.

### Concluding remarks

The original intention for this work was to locate the site of a potential novel variant, causing perinatal mortality in Irish Moiled calves. This has been achieved, and shown to be located in the *GCK* gene, but in the process it became apparent that there were no straightforward ways to achieve this objective. At best, a two-stage approach was required, involving genotyping a group of cases and controls, identifying the genomic region likely to contain the novel variant followed by further work to sequence the identified region and look for appropriate signals in the data. Consequently, a further objective was set in order to simplify the process and investigate whether it would be possible to use a single whole genome sequencing stage with appropriate bioinformatic methodology to find the candidate site. This too has been achieved by sequencing nine animals, three cases, six parental controls, and applying four methods to the data. In the process, it has been possible to investigate some of these methods in more detail and arrive at some general conclusions to aid future such studies.

The VCF file format has proven to be a very practical source of data for this study particularly because it reduced the search “area” from over 2.5 billion base positions down to one involving only 9 million sites. In addition, the VCF file format facilitated finding the novel site when combined with methods to interrogate it for genotype criteria, long runs of homozygosity, and the predicted effects of variants on the phenotype of the animal. Using these three methods allowed the identification of a single variant site, which was found to have both the genomic and biological properties associated with this novel condition.

In the process of carrying out this work it has been possible to refine the genotype criteria method to demonstrate that in reality only a small number of cases and controls *are* required, and controls should outnumber cases by 2:1 and controls should be more distantly related to cases. In addition, it has been possible to show that using a runs-of-homozygosity method, previously used only on SNP-chip genotype data with whole genome sequence data, it was possible to locate the region of the genome containing the novel variant.

In future it should be possible to use the combination of genotype criteria and runs of homozygosity methods with the appropriate number of cases and controls, suitably distantly related, to locate the site of any new autosomal recessive genetic condition in a relatively short time. This should then facilitate a more speedy elimination of the harmful variant from the population by using an appropriate genetic test on available animals.

## Data Availability

The datasets presented in this study can be found in online repositories. The names of the repository/repositories and accession number(s) can be found below: https://rvc-repository.worktribe.com/output/1551650.
