# Supplementary material for: Locating a novel autosomal recessive genetic variant in the cattle glucokinase gene using only WGS data from three cases and six carriers
Source: Front Genet. 2022 Aug 29;13:755693. doi: 10.3389/fgene.2022.755693 (PMC9465091; doi:10.3389/fgene.2022.755693)
Supplement: Supplementary file 1 [file DataSheet1.docx]

Supplementary text for:

**Locating a novel autosomal recessive genetic condition using only WGS data from three cases and six controls; a case study of a new variant in the cattle glucokinase gene**

By Geoffrey Pollott, Richard Piercy, Claire Massey, Mazdak Salavati, Zhangrui Cheng and D. Claire Wathes

**CONTENTS:**

**METHODS**

Whole-genome sequence DNA extraction Page S2

Alignment, mapping and variant calling Page S2

Hard-filtering of the VCF file Page S4

Figure S1 The distribution of the VCF file site-level quality measures Page S4

Figure S2 The distribution of the VCF file genotype-level quality measures Page S5

Table S1 Hard-filtering levels applied to the final VCF file to select SNPs

for the ABD analysis Page S6

Figure S3 Pedigree relationships between the WGS animals Page S6

**RESULTS**

Figure S4 Manhattan plots of ROH for the nine case/control animals from the

WGS data (Kb) Page S7

Table S2 Summary for the regions found by ABD with P < 0.001 Page S8

Table S3 A summary of sites with no registered RS number Page S8

Figure S5 Manhattan plot of ROH scores for cases/controls; SNP data (Kb) Page S9

**DISCUSSION**

An investigation into using the genotype criteria method

*1) The distribution of genotype criteria sites across the genome* Page S10

*2) The effect of the number of animals genotyped on finding GCR sites* Page S13

*3) The balance of cases and controls* Page S15

4) *The genetic relationship between cases and controls* Page S16

A study into the implied mode of inheritance from a farm survey Page S18

**METHODS**

**Whole-genome sequence DNA extraction**

Tufts of tail hairs from both adult cows and bulls and dead calves were posted to the laboratory Working in a laminar flow hood under sterile conditions, 5-10 mm lengths including the hair follicle were cut individually from the base of each of about 20 hairs per animal and transferred into a 1.5 ml microcentrifuge tube.

DNA was extracted from hair samples using a DNeasy Blood and Tissue Kit (Qiagen) based on the supplied protocol with minor modifications. Briefly, to lyse the hair samples, they were first incubated with 300 µl buffer ATL, 20 µl proteinase K and 20 µl 1M dithiothreitol (Sigma) at 56 °C overnight while shaking. Next 300 µl buffer AL and ethanol (96-100%) were added and fully mixed. The sample was loaded onto the DNeasy Mini spin column and centrifuged at ≥ 6000 × g_n_ for 1 minute at room temperature. The flow-through was discarded and the column was washed with 500 µl Buffer AW1 and centrifuged for 1 min at ≥6000 x g_n_. Next 500 μl Buffer AW2 was added followed by centrifugation for 3 min at 20,000 x g to dry the DNeasy membrane. To collect the DNA, 200 μl Buffer AE was pipetted onto the column, incubated at room temperature for 5 min, and then centrifuged for 1 min at ≥6000 x g_n_ to elute. The DNA concentration and 260/280 ratios were quantified with a NanoDropND-1000 spectrophotometer (NanoDrop Technologies Inc., Wilmington, DE, USA) and samples were then stored at –80 °C.

**Alignment, mapping and variant calling**

Raw fastq files were trimmed and quality controlled using Trim_Galore v.0.4.5 given the following flags: -q 26 --paired (Cutadapt v1.15 (http://code.google.com/p/ cutadapt/ ; Andrews, 2010). Trimmed fastq files were then mapped against the UMD3.1.1 assembly (ENSEMBl v87) using a Burrows-Wheeler aligner (BWA; Li and Durbin, 2009) given the following flags: bwa mem -t 64 -M. BWA output was converted and sorted into BAM files using Samtools (Li et al., 2009) followed by a GATK best practice workflow for variant calling (McKenna et al., 2005; Van der Auwera et al., 2013). PCR duplicates were marked (Picard tools MarkDupilcates) and the following steps were carried out using GATK tools (Broad Institute, 2020). In all steps the reference genome (-R) and variant annotation files (known) were the ENSEMBL v87 fasta and VCF files:

1. Indel Target Intervals (GenomeAnalysisTK - T RealignerTargetCreator -nt 64 -R -known)

2. Indel realignment of BAM files (GenomeAnalysisTK -T IndelRealigner -R -targetIntervals -known)

3. Illumina Base Quality recalibration (GenomeAnalysisTK -T BaseRecalibrator -nct 64 -R -knownSites)

4. Printing the BQSR reads (GenomeAnalysisTK -T PrintReads -nct 64 -R -BQSR)

5. Haplotype calling while emitting all nucleotide (GenomeAnalysisTK -T HaplotypeCaller -ERC gVCF --stand_call_conf 30)

6. Joint genotyping of gVCF call set (GenomeAnalysisTK -T GenotyeGVCFs --dbsnp ).

7. The nine individual vcf files were merged into one final vcf file using bcftools logic set to accept only biallelic SNPs and indels.

8. Single nucleotide variants were filtered using GATK v3.8 (REF PMID: 25431634) best practice hard filtration guidelines. The following excluding flags were used: QD < 2 (quality by depth), QUAL <30 (variant Phred score quality), SOR >3 (strand bias probability), FS>60 (Strand bias tested by Fisher), MQ <40 (mapping quality of the originating reads), MQRankSum < -12.5 ( MQ rank sum test statistics), ReadPosRankSum <-8 (MQ rank sum for

9. A coordinate liftover was carried out from the UMD3.1 to ARS-UCD1.2 genome on the final VCF file using CrossMap (Zhao et al., 2013).

**References**

1. Andrews, S. (2010) FastQC a Quality Control Tool for High Throughput Sequence Data [Online], Babraham. <http://www.bioinformatics.babraham.ac.uk/projects/fastqc/>.

2. Li, H., and Durbin, R. (2009). Fast and accurate short read alignment with Burrows-Wheeler transform. Bioinform. (Oxford, England) 25, 1754–60.

3. Li, H., Handsaker, B., Wysoker, A., Fennell, T., Ruan, J., Homer, N., Marth, G., Abecasis, G., and Durbin R. (2009) 1000 Genome Project Data Processing Subgroup (2009) The Sequence Alignment/Map format and SAMtools. Bioinform. 25, 2078–2079.

4. McKenna, A., Hanna, M., Banks, E., Sivachenko, A., Cibulskis, K., Kernytsky, A., Garimella, K., Altshuler, D., Gabriel, S., Daly, M., and DePristo, M.A. (2010) The Genome Analysis Toolkit: a MapReduce framework for analyzing next-generation DNA sequencing data. Genom. Res. 20, 1297–303.

5. Van der Auwera, G.A., Carneiro, M.O., Hartl, C., Poplin, R., Del Angel, G., Levy-Moonshine, A., Jordan, T., Shakir, K., Roazen, D., Thibault, J., Banks, E., Garimella, K.V., Altshuler, D., Gabriel, S., and DePristo, M.A. (2013) From FastQ data to high confidence variant calls: the Genome Analysis Toolkit best practices pipeline. Curr. Protoc. Bioinform. 43, 1-33.

6. Broad Institute. (2020) The Broad Institute, Cambridge, USA. <https://gatk.broadinstitute.org/hc/en-us>. [Accessed October 21st, 2020]

7. Zhao, H., Sun, Z., Wang, J., Huang, H., Kocher, J.-P., and Wang, L. (2013). CrossMap: a versatile tool for coordinate conversion between genome assemblies. Bioinformatics (Oxford, England), 30, 1006-7. doi: 10.1093/bioinformatics/btt730.

**Hard-filtering of the VCF file**

**Figure S1 The distribution of the VCF file site-level quality measures**


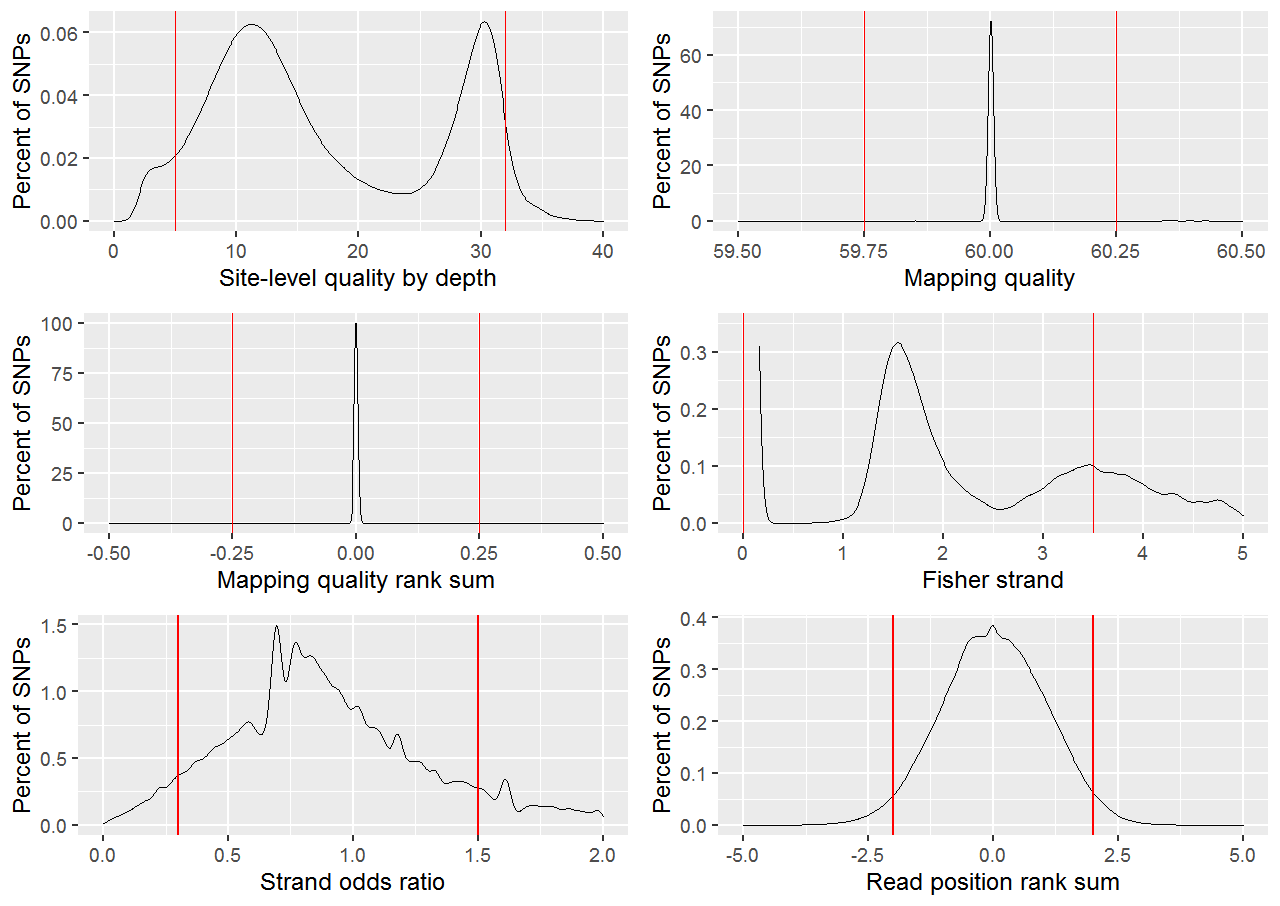


Legend: These six site-level measures were used in the hard-filtering process to calculate ABD scores from the WGS data. The sites were selected by applying a limit of the mean ±2 s.d. for normally distributed traits and the appropriate lower or upper bound for the normally distributed components of bimodal traits or single-tailed traits. So for example, read position rank sum was normally distributed so the mean ±2s.d. was used as the hard-filtering cut-offs. For site-level quality by depth, which was bi-modal, the lower peak -2s.d. was used as the lower cut-off and the upper peak +2s.d. as the upper cut-off. A value of 0 is a good score for the Fisher strand quality so all values below the upper 2s.d. level were used. See Table 1 legend for units.

**Figure S2 The distribution of the VCF file genotype-level quality measures**

**
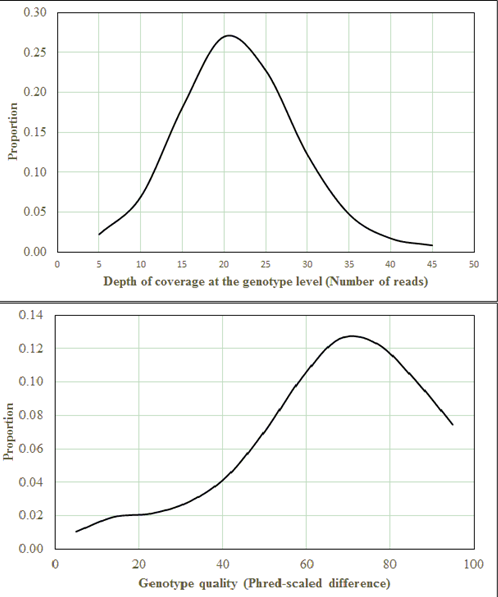
**

Legend: These two genotype-level measures were used in the hard-filtering process to calculate ABD scores from the WGS data. The sites were selected by applying a limit of the mean ±2 s.d. for read depth (GDP) and since the genotype Phred-scaled quality score (GQ) assigned a value of 99 to all values above 99 there is only a lower bound for that measure.

**Table S1 Hard-filtering levels applied to the final VCF file to select SNPs for the ABD analysis**

| **Quality measure (site or genotype level)** | **Abbreviation** | **Filter levels** |
| --- | --- | --- |
| Quality by depth (site) | QD | >5 or <32 |
| Mapping quality (site) | MQ | >59.75 or <60.25 |
| MQ rank sum (site) | MQRS | >-0.25 or <0.25 |
| Fisher strand (site) | FS | <3.5 |
| Strand odds ratio (site) | SOR | >0.3 or <1.5 |
| Read position rank sum (site) | RPRS | >-2 or <2 |
| Read depth (genotype) | GDP | >12 or <28 |
| Phred-scaled quality (genotype) | GQ | >54 |

Legend: Filter levels refer to the sites or genotypes retained in the dataset. QD-Ratio of quality to unfiltered depth of non-referenced samples; MQ-Root-mean-square of the mapping quality of the reads across all samples; MQRS-The u-based z-approximation from the Mann-Whitney Rank Sum test for mapping quality; FS-Phred-scaled p value from Fisher’s exact test to detect strand bias in reads; SOR-Log-scaled allele-specific strand bias estimated by the Symmetric Odds Ratio test; RPRS-The u-based z-approximation from the Mann-Whitney Rank Sum test for the distance from the end of the read for reads with the alternate allele; GDP-Number of reads; GQ-Conditional genotype quality, encoded as a Phred quality score -10log_10_P.

**Figure S3 Pedigree relationships between the WGS animals**


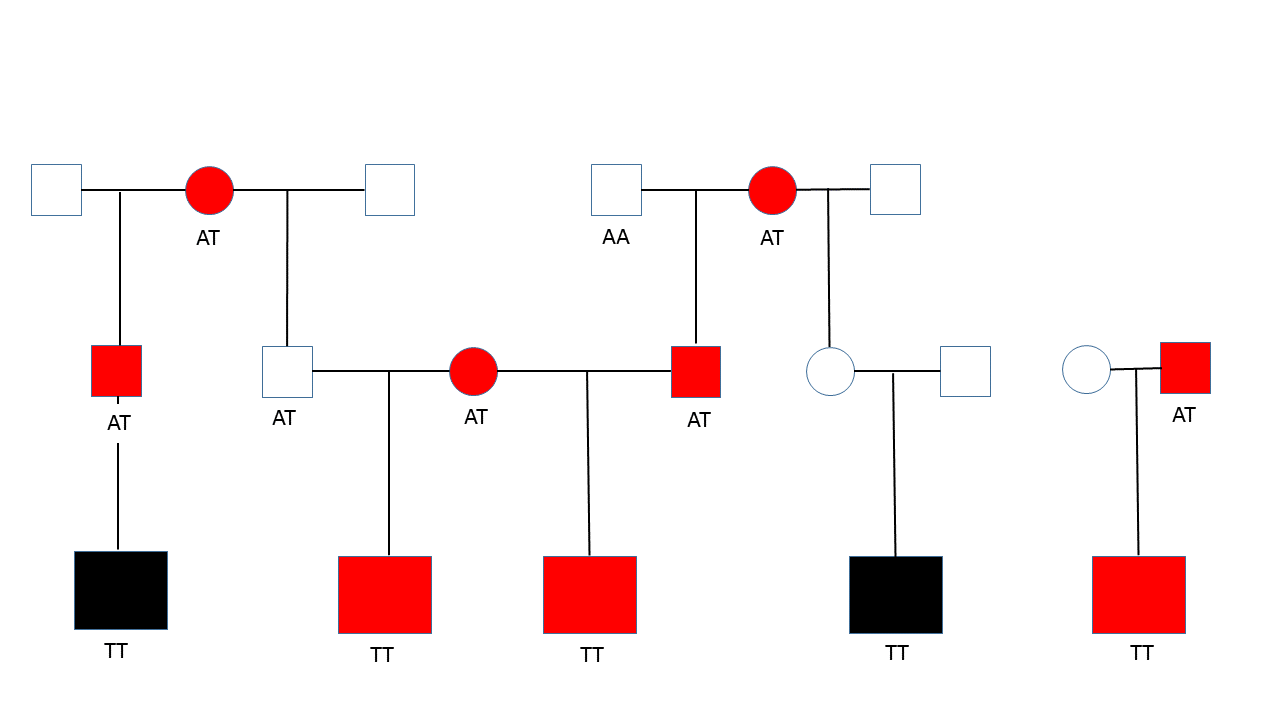


**Legend:** Pedigree relationships for five dead calves with known TT genotypes (rectangles at base of figure) and all nine WGS animals (red infill). Genotypes shown are from either WGS or PCR results. Circles and squares in the top two lines indicate males and females respectively.

**RESULTS**

**Figure S4 Manhattan plots of ROH for the nine case/control animals from the WGS data (Kb)**

**
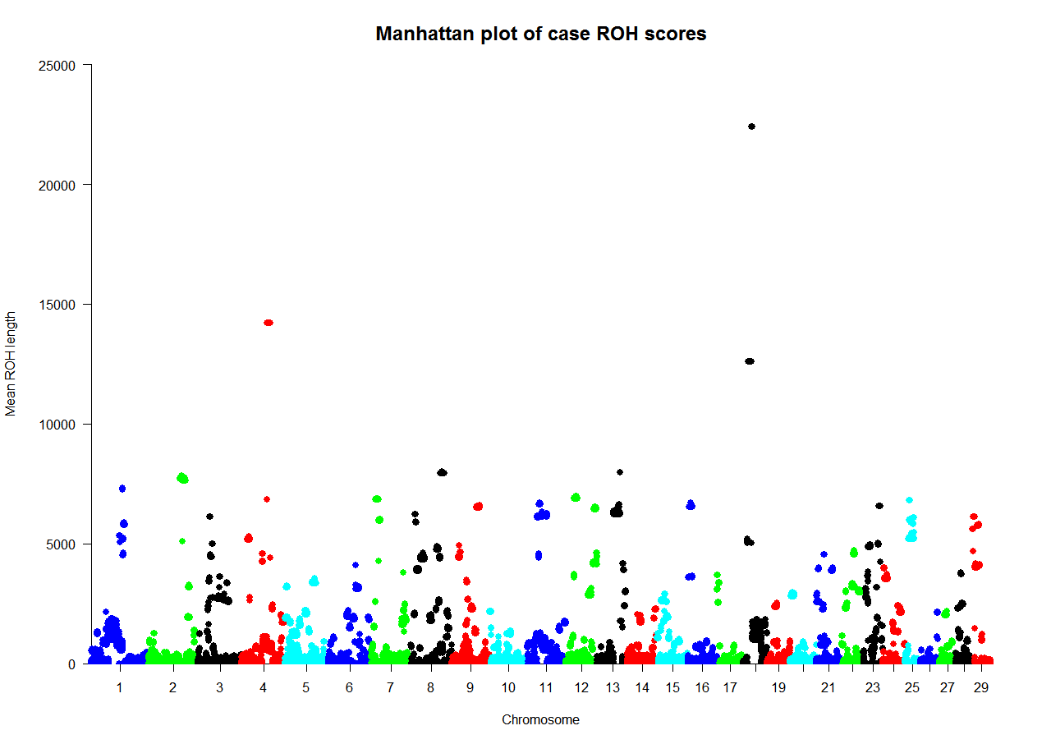
**

**
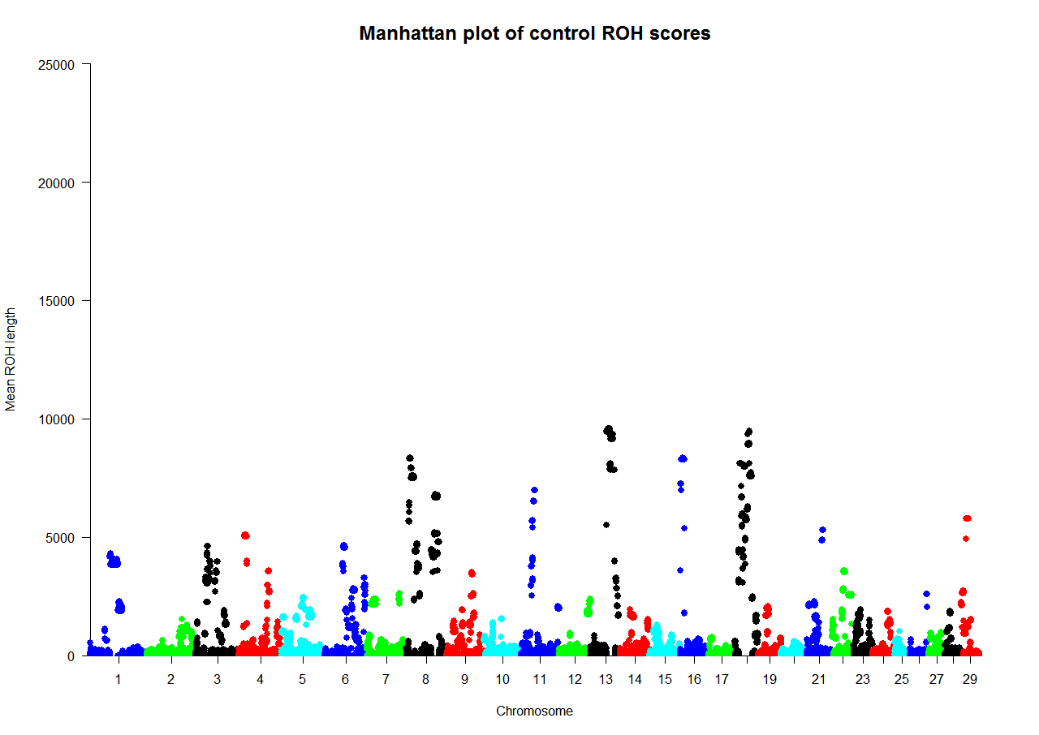
**

**Table S2 Summary for the WGS regions found by ABD with P < 0.01**

| **BTA** | **Start position** | **End position** | **Length (Mb)** | **No. of SNV^1^** |
| --- | --- | --- | --- | --- |
| 4 | 70,889,821 | 78,693,399 | 7.803 | 18,705 |
| 18 | 23,056,293 | 25,152,359 | 2.096 | 6,618 |

Legend:^1^Number of SNV in this length of the chromosome from the final VCF file

**Table S3 A summary of sites with no registered RS number**

| **Number of genotypes containing the variant allele** | **Number of sites** | **% of sites** |
| --- | --- | --- |
| 1 | 168,691 | 49 |
| 2 | 62,258 | 18 |
| 3 | 37,667 | 11 |
| 4 | 23,012 | 7 |
| 5 | 18,538 | 5 |
| 6 | 12,100 | 4 |
| 7 | 8,390 | 2 |
| 8 | 7,869 | 2 |
| 9 | 6,372 | 2 |
| Total | 344,898 |  |

Legend: Results shown by the number of genotypes at that site containing the SNV or indel.

**Figure S5 Manhattan plot of ROH scores for cases/controls; SNP data (Kb)**


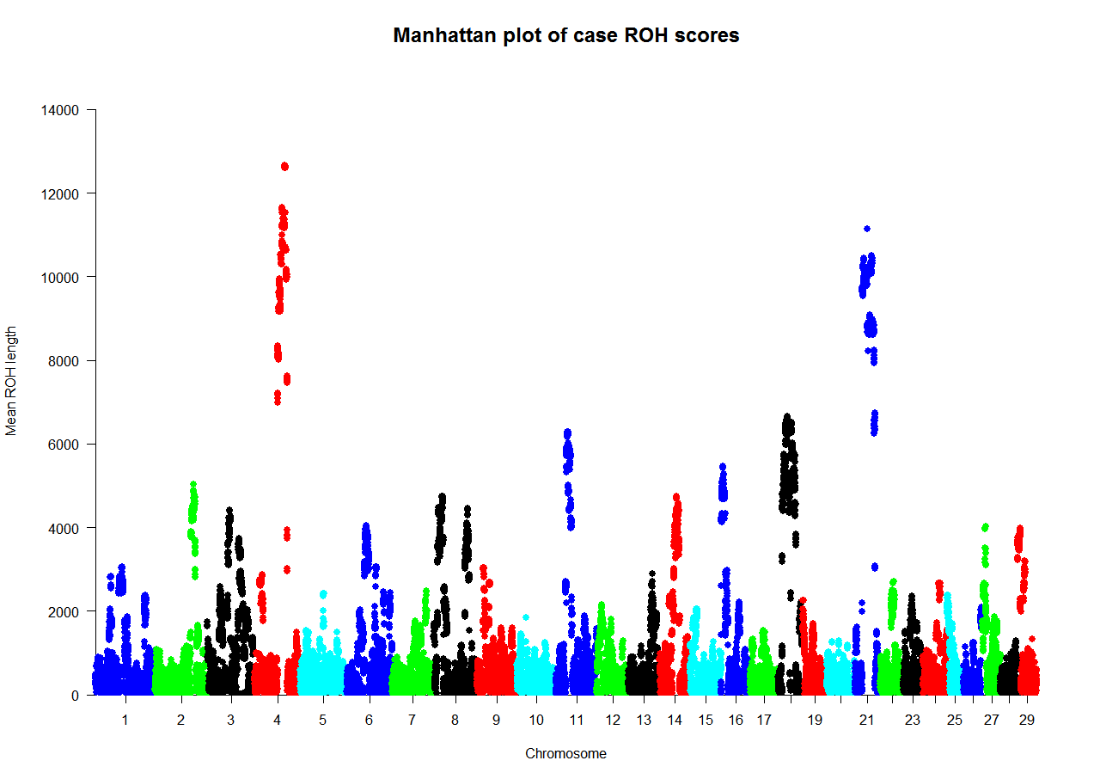


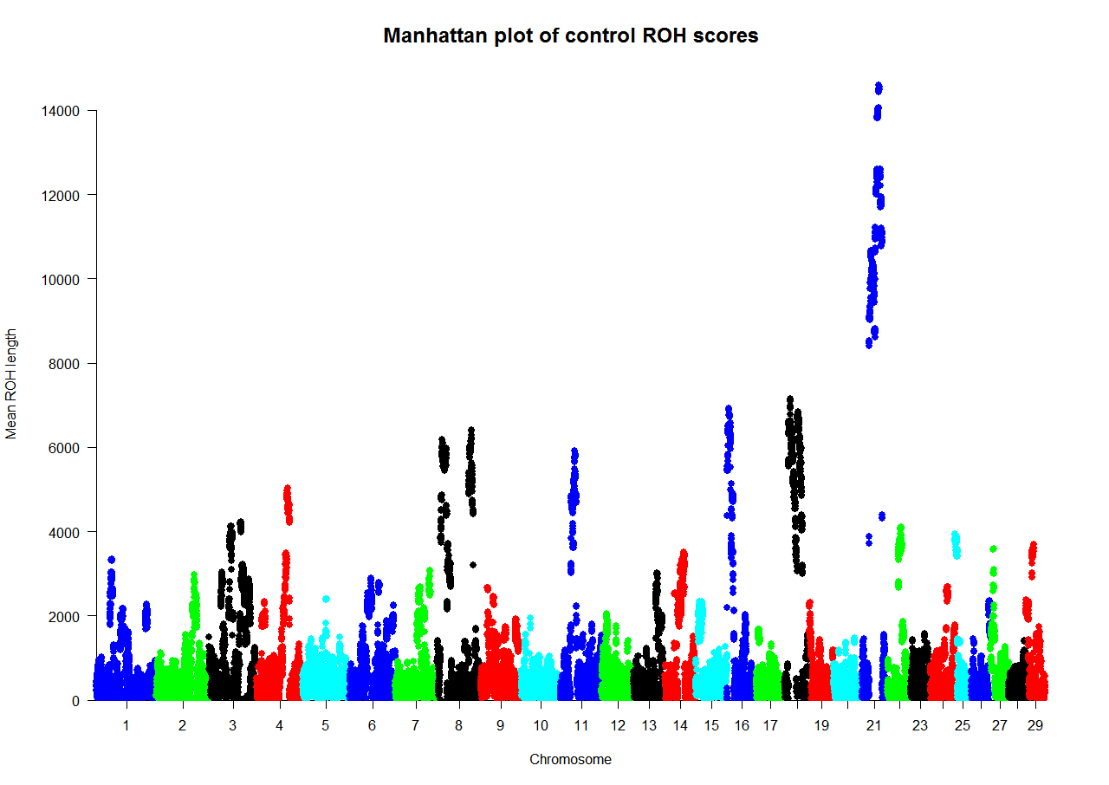


**DISCUSSION**

**An investigation into using the genotype criteria method**

*1) The distribution of genotype criteria sites across the genome*

BTA4 was investigated in more detail to illustrate the distribution of sites with six heterozygous genotypes in controls, and also those sites with three homozygous variant genotypes in cases using the final VCF file (i.e. unfiltered). Figure S6 shows the distribution of sites with six heterozygous controls along BTA4 by 1Gb windows. They do not appear to be evenly spread along the chromosome. The distribution of cases with three variant homozygous genotypes is shown in Figure S7. This shows a more even distribution across the chromosome than seen for the heterozygotes with the expected peak around the region containing the new variant site. The two sets of data are combined in Figure S8, along with the sites meeting the genotype criteria (GCR). There is an indication that there may be an inverse relationship between the two components of the genotype criteria (all heterozygous controls and all homozygous variant cases).

**Figure S6 The distribution of sites with six heterozygous genotypes (0/1) in controls on BTA4. The region found to contain the candidate site by ABD was between base positions 70 and 80 million***.*


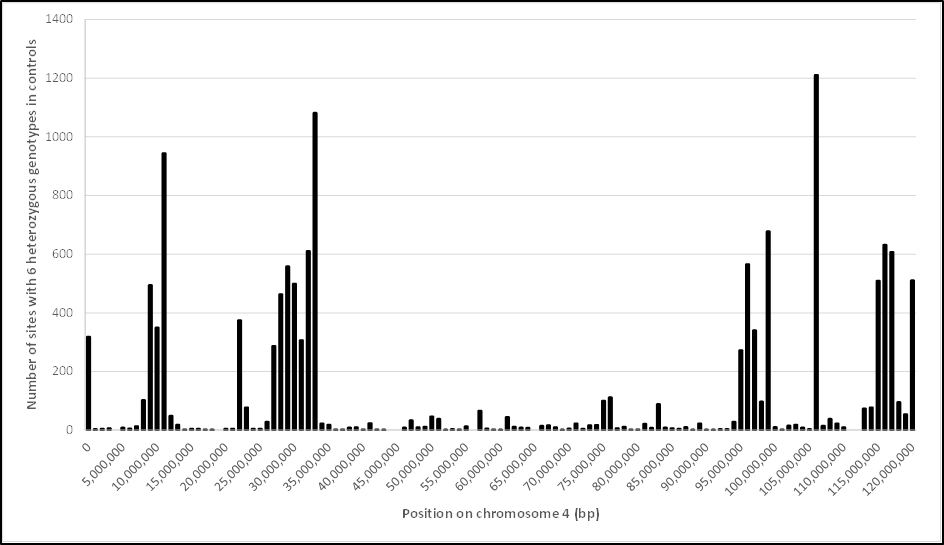


**Figure S7 The distribution of sites with three homozygous variant genotypes (1/1) in cases on BTA4. The region found to contain the candidate site by ABD was between base positions 70 and 80 million.**

**Figure S8 The distribution of sites with six heterozygous genotypes and those with three homozygous variant genotypes on BTA4 (left-hand axis), including the number of genotype criteria sites (in red and right-hand axis). The region found to contain the candidate site by ABD was between base positions 70 and 80 million.**


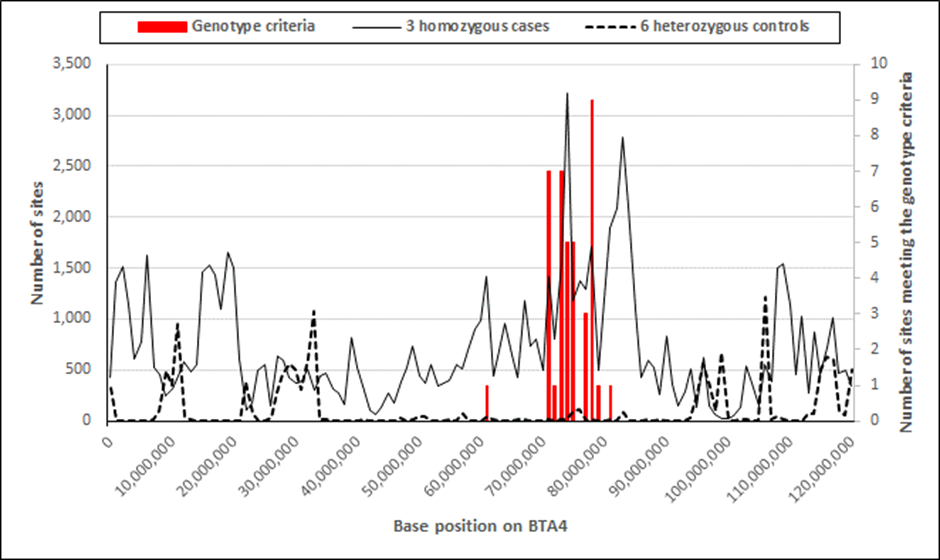


Further investigation using regression analysis, however, did not find a strong relationship between the two sets of data (Figure S9). The correlation between the number of sites within each 1Gb window was 0.17, although the line of best fit using a polynomial indicates a slight negative relationship. The regions with a high incidence of all-homozygote cases tended to have low numbers of all-heterozygous controls (0-100 per Gb region) whereas the regions with high numbers of all-heterozygous controls was spread over a range of values for sites with all-homozygous cases (0-1000 per Gb region).

Considering the length of the region with high ROH scores on BTA4 in relation to the chromosome’s length there are virtually no GCR sites outside the long-ROH region and they are certainly not spread evenly along the rest of the chromosome. If these results are indicative of the genomewide situation then we would not expect the distribution of GCR sites to be evenly spread throughout the genome but concentrated in areas of high homozygosity.

**Figure S9 A plot of the relationship between the two components of the genotype criteria from VCF data on BTA4. Line of best fit shown as a second-order polynomial**


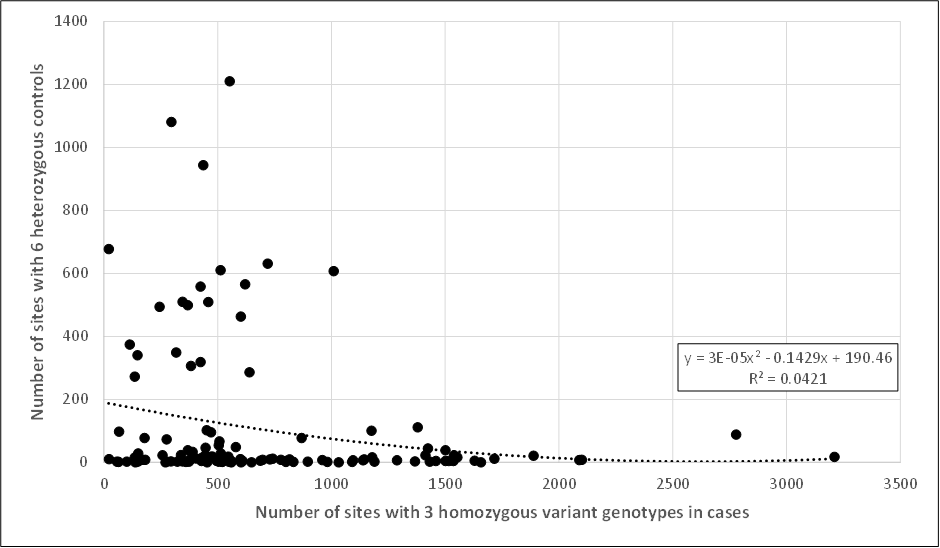


Table S4 shows the distribution of GCR sites and the average ROH length in which they were found for the GCR sites reported in Table 1 along the complete genome. The candidate chromosome (BTA4) and BTA1 had a high mean ROH length but the other chromosomes contained GCR sites with a mean value ranging from 0 to 8,509 Kb. Average ROH values of 0 indicate GCR sites were single sites not in a ROH. Clearly there is very little in the way of general conclusions about the distribution of GCR sites throughout the genome other than the largest groups are found in long ROH and that the sites with all-heterozygous genotypes in controls were not spread evenly throughout the genome but appear in clusters.

**Table S4 The distribution of genotype criteria sites by chromosome and the average length of ROH in cases in which they were found.**

| BTA | Number of genotype criteria sites | Average case ROH containing the genotype criteria sites (Kb) |
| --- | --- | --- |
| 1 | 582 | 11,411 |
| 2 | 4 | 6 |
| 4 | 22 | 19,402 |
| 5 | 2 | 4,275 |
| 6 | 4 | 38 |
| 7 | 2 | 967 |
| 8 | 3 | 3,555 |
| 9 | 2 | 6,652 |
| 10 | 1 | 3,553 |
| 11 | 9 | 2,150 |
| 12 | 21 | 198 |
| 13 | 2 | 573 |
| 14 | 1 | 2,663 |
| 15 | 2 | 206 |
| 17 | 5 | 5,005 |
| 20 | 1 | 151 |
| 21 | 2 | 3,883 |
| 22 | 1 | 5,597 |
| 23 | 58 | 3,967 |
| 24 | 3 | 825 |
| 26 | 1 | 0 |
| 27 | 1 | 0 |
| 28 | 1 | 35 |

*2) The effect of the number of animals genotyped on finding GCR sites*

Figure S10 shows that the theoretical number of animals required to find the candidate site underestimates the number found ‘in practice’. This was further investigated using the SNP dataset with the candidate site identified by PCR. Forty-two animals (12 cases and 30 controls) were used for these analyses. Different combinations of cases and controls were randomly selected to see how the variability of the number of GCR sites found changed with both a different ratio of cases to controls and also a differing overall number of animals used. This was repeated 100 times (i.e. with randomly selected groups of animals each time) for each combination of cases and controls used. Table S6 shows one set of results for four cases and a variable number of controls. The 95% confidence interval is shown for this table in Figure S11.

**Figure S10 A summary of several sets of samples of the SNP data showing how the mean number of genotype criteria sites found varies by the number of cases (and controls) used compared to the theoretical number. (Logarithmic scale on y axis)**

**Figure S11 The mean number of genotype criteria sites found by the number of animals genotyped using 100 samples of four cases and a variable number of controls. The 95% confidence interval is shown above and below the mean line (black). (Logarithmic scale on y axis).**

**Table S5 Mean and standard deviation of the number of GCR sites found by randomly selecting four cases and varying number of controls from the SNP dataset.**

| Number of controls | Total number of animals selected | Mean number of GCR sites found | SD of the number of GCR sites found | CV of the number of sites found |
| --- | --- | --- | --- | --- |
| 1 | 5 | 1,451.1 | 699 | 48 |
| 5 | 9 | 26.4 | 14 | 53 |
| 9 | 13 | 7.2 | 6 | 84 |
| 13 | 17 | 3.3 | 2 | 89 |
| 17 | 21 | 2.1 | 1 | 80 |
| 21 | 25 | 1.5 | 1 | 86 |
| 25 | 29 | 1.4 | 0 | 58 |
| 29 | 33 | 1.0 | 0 | 16 |

As the number of animals genotyped increases the number of GCR sites found falls until, using 33 animals leaves only one site, the candidate site, as expected. However, the variability of the 100 randomly selected samples of the SNP data is high in relation to the mean number of sites found. Figure S11 illustrates that, in this example, for about nine or more animals genotyped some samples may find the only one site meeting the genotype criteria but on the other hand they could find anything up to 55 sites. Hence the variability is high.

*3) The balance of cases and controls*

Figure S10 shows a summary of several sets of sampled data (100 repetitions) with different combinations of cases and controls plus the theoretical ‘expected’ number of GCR sites for each combination of cases and controls used.

Initially, using more animals reduces the number of potential GCR sites found. However, as the number of animals used increases there is a dropping off in the rate of decline in GCR sites found.

Figure S10 demonstrates that the lower the number of cases used the fewer the total number of animals required to be genotyped. At first sight these are rather startling results. However, outside the candidate site, it is much more unlikely to find all controls with a heterozygote genotype, whereas there will be many sites with all homozygous cases; after all long ROH imply many 1/1 genotypes and so more sites potentially could meet the genotype criteria. This reflects the data presented earlier from the BTA4 VCF file.

The above analyses were rerun but excluding the two regions of long ROH shown in Figure S5 (cases). The expectation was that the data would be closer to the theoretical numbers in the ‘non-autozygous’ regions of the genome. The results are shown in Figure S12. They clearly demonstrate that the number of animals required is much closer to the theoretical numbers than shown in Figure S11. The number of animals required is still slightly greater but this may be due to some other small areas of ROH not removed from the dataset. There are an enduring number of GCR sites in the high ROH regions which inflate the results in contrast to the theoretical number of sites expected. This illustrates why the theory and practice may differ.

**Figure S12 A summary of several sets of samples of the SNP data showing how the mean number of genotype criteria sites found varies by the number of cases (and controls) used compared to the theoretical number, excluding two regions of long ROH. (Logarithmic scale on y axis)**

4) *The genetic relationship between cases and controls*

In order to investigate whether the closely-related controls might inflate the number of GCR sites found, an alternative SNP-chip dataset was derived using controls which were most distantly related to the cases. The relatedness coefficients derived from the KING analysis were used to find a group of 10 cases with the lowest relationship to a group of 12 non-parental controls. The analyses described above were rerun on this smaller set of data and the results summarised in Figure S13.

In this case the number of animals required to find the new variant site was much closer to the theoretical expectation than with the parental controls. The main reason for this was the lower number of GCR sites in the long ROH region (found in cases) due to fewer all-0/1 (and 0/0) sites found in controls. Note that when using more distantly related individuals then the GCR calculation is 2/(3^n^x3^m^) but this hardly affects the expected number of animals required due to the large denominator.

**Figure S13 A summary of several sets of samples of the SNP data showing how the mean number of genotype criteria sites found varies by the number of cases (and controls) used compared to the theoretical number, using more distantly related individuals. (Logarithmic scale on y axis)**

Figure S13 shows that when using five to nine cases only 14 to 17 animals need to be genotyped in total to find the one GCR site. This implies that about 33 to 50% of samples need to be cases in order to get a good result.

**A study on the implied mode of inheritance from a farm survey**

During the summer of 2008 herds of Irish Moiled cattle were visited and data were collected on as many calvings as possible. Three basic records were collected on 28 farms; a cow record, a bull record and farm information. The data reported here were the calvings recorded against each cow with particular care being taken to record the fate of unregistered calves. Since there was no definitive test available to categorise the early calf deaths they were divided into three groups; Type 1 – a likely death due to the novel condition under study, Type 2 – a death possibly due to the condition and Type 3 – an unrelated death. Two sources of pedigree information were used in these analyses. Data collected on farm included the sire and dam of each calf recorded. In addition files were obtained from the Irish Moiled Breed Society and merged with the data collected on farm, cross-checking being undertaken to ensure consistency of both registration identity and animal name in all instances of their uses.

The nature of this early calf death condition suggested that it was either genetic in origin or confined to specific farms and therefore a management/’environmental’ issue on those farms. Since the occurrence of the condition had been only reported from Irish Moiled herds this suggested that it is most likely to be genetic in origin. This possibility was tested by carrying out a statistical analysis of the occurrence of the condition to investigate whether farm, sire, sex of calf or year of birth could explain some of the variation in its occurrence. An analysis of variance was undertaken using a binomial distribution for the trait of interest, the occurrence or not of the condition in a calf.

*Binomial model*

The calf mortality data from the farm survey were analysed by generalized linear model using a binomial link function in ASReml (Gilmour et al., 2009). The model fitted was :

Y_ijklm_ = µ + f_i_ + s_j_ + y_k_ + sr_l_ +e_ijklm_

where Y_ijklm_ was the live/dead status of the calf, µ the overall mean, f_i_ was the ith farm of birth of the calf, s_j_ was the jth sex of calf, y_k_ was the kth year of birth, sr_l_ was the lth calf sire and e_ijklm_ was the random error term. The probability of an effect was tested against the error variance on the underlying binomial scale using an F ratio.

*Survey of breeders*

The data collected from 28 farms comprised 881 calves (395 males, 455 females, 12 castrates and 19 unknown sex), the offspring of 84 sires and 229 dams. There were 1,777 animals in the pedigree file (i.e. all animals and their ancestors, as far back as possible) developed from both the Society’s records and data collected on farms. A summary of all calf deaths is shown in Table 1.

**Table 1. The incidence of calf deaths in the 28 recorded herds.**

|  | **Type 1*** | **Type 2** | **Type 3** | **Total** |
| --- | --- | --- | --- | --- |
| **Number** | 14 | 21 | 38 | 72 |
| **% of deaths** | 19.4 | 29.2 | 51.4 |  |
| **% of calves** | 1.6 | 2.4 | 4.2 | 8.2 |
| **Males** | 7 | 6 | 14 | 27 |
| **Females** | 5 | 12 | 13 | 30 |
| **Unknown sex** | 2 | 3 | 11 | 16 |

* Type 1 = probable cases; Type 2 = possible cases; Type 3 = unrelated deaths.

There were 14 affected animals (cases; 7 males, 5 females and 2 unknown sex), the offspring of matings between 7 sires and 10 dams.

**Analysis of variance results**

The results of applying Model 1 in a generalized linear model on the incidence of the Type 1 deaths within and between families are shown in Table 2.

**Table 2. An analysis of variance of the occurrence of early calf death in Irish Moiled cattle.**

| **Source of variation** | **DF** | **F** | **Probability** |
| --- | --- | --- | --- |
| **Sire** | 83 | 1.45 | 0.041 |
| **Year** | 14 | 0.41 | 0.969 |
| **Farm** | 28 | 1.12 | 0.315 |
| **Calf sex** | 3 | 0.18 | 0.908 |

Table 2 indicates that the between sire family effect was significant (P < 0.05) which means that the condition ran in sire families and therefore is likely to have a genetic basis. There was no significant effect of year, farm or sex on the condition once the effect of the sire was considered. This implies that the condition does not depend on the farm where the calf was born, over and above the effect of its sire. Thus it is not the farm (animal management, health regime, feeding) itself causing the condition. Further evidence for this arises because the condition has not been reported in other breeds on other farms, or in other animals on the same farms either. Also the condition is not related to the sex of the calf.

**Mode of inheritance**

Considering the possible single-gene modes of inheritance in relation to the data summarised above, the following conclusions may be drawn about the genetic models of inheritance describing the data. A dominant model is not possible with an early onset fatal condition. It is also unlikely to be a sex-linked condition since there was no effect of sex on the incidence of the condition. Thus it is most likely to follow the autosomal recessive mode of inheritance.

If the condition is due to an autosomal recessive gene then both parents of an affected calf must be carriers of the gene. Since the 14 affected calves were the offspring of 7 sires and 10 dams we can identify 17 known carriers. The pedigrees of these 17 known carriers were analysed to see if they had any ancestor in common which might suggest a point of origin of the condition. All 17 pedigrees did lead back to a common sire, long since dead. This bull is thus implicated as either the originator of the condition in the Irish Moiled population, or is a direct descendant of the originator and no carriers identified in this survey led back to that possible other originator. This second possibility might occur if this bull’s dam was the originator and, by chance, she left no other carriers. It would be possible to be more precise about the exact originator if more calves dying of the condition could be identified. Further analysis of the pedigrees identified another 11 animals (4 males and 7 females) who must be carriers since they formed unambiguous links between the known carriers and the suspect bull.

The only likely Mendelian model to explain the current data was the autosomal recessive model. Normally this could be tested by comparing the expected and observed incidence of the condition in known carrier matings. The 22 of the 28 known or implied carriers produced 30 offspring when mated together with the expectations shown in Table 3. These were calculated from the 3:1 (unaffected : affected) Mendelian ratio expected from the mating of two heterozygotes applied to the 30 offspring found in the data. The statistical analysis of this table implied that incidence of calf deaths amongst heterozygote matings did not conform to the 3:1 expected ratio. In Table 3 it can be seen that there were more affected animals that expected. This may be due to the survey concentrating on herds where affected herds were thought to have occurred. Also there was a low incidence of known carrier/carrier matings in the population which may have affected the results. In addition, later genotyping of a sample of the breed indicated that there was a tendency for breeders to submit any dead calf as a likely case. This would inflate the ‘affected’ numbers and reduce the ‘unaffected in Table 3.

**Table 3. The observed and expected incidence of the condition in the families of known and implied carriers.**

|  | Observed | Expected |
| --- | --- | --- |
| Affected | 14 | 7.5 |
| Unaffected | 16 | 22.5 |
